# A harmful traditional practice exposing young girls to experience virgin pregnancy (*Shilshalo*): a qualitative study in Argoba community, Amhara National Regional State, Ethiopia

**DOI:** 10.1186/s12914-018-0179-x

**Published:** 2018-11-20

**Authors:** Yohannes Mersha Belete, Negesse Kebede Atlaw

**Affiliations:** 1 Department of Gender and Development Studies, Bahir Dar University, Bahir Dar, Ethiopia; 2 Gender and Health Expert, Addis Ababa City Administration Rehabilitation Project Office, Addis Ababa, Ethiopia

**Keywords:** *Shilshalo*, Harmful traditional practice, Girls, *Argoba* community, Ethiopia

## Abstract

**Background:**

There are various harmful traditional practices and beliefs across the different parts of Ethiopia. *Shilshalo,* which is yet little known about, is one of these practices existing in *Argoba*, a community in Amhara National Regional State of Ethiopia. This study was conducted to explore the various features associated with the practice of *Shilshalo.*

**Methods:**

To address the objective of the study, qualitative approach with case study design was employed. Purposive sampling technique was used to select the participants of the study. Data were collected through interview and focus group discussion and analyzed using thematic qualitative analysis technique.

**Results:**

This study found that *Shilshalo* is practiced by unmarried young boys and girls as a substitute for sexual intercourse. The actors conduct all activities performed during sexual intercourse except inserting the boy’s genital organ (penis) into the girl’s (vagina). The activities include warming up the girls’ body by hand, kissing and brushing the girl’s thigh and the areas around the outer part of the vagina with the erected penis. *Shilshalo* is practiced in two ways, i.e. between a boy and a girl, and between boys and a girl. This study also indicated that most members of the *Argoba* community including the actors consider *Shilshalo* as a beneficial cultural practice, yet the most shocking story is that some teenage girls experienced unwanted pregnancy without losing their virginity. In addition to virgin pregnancy, *Shilshalo* exposed girls to STIs, psycho-social problems and physical injuries.

**Conclusion:**

This study concluded that *Shilshalo* is a harmful traditional practice that severely affects the lives of girls. The overall health and social well beings of girls are affected by different saddles that came from it. It brings almost all kinds of consequences that the other harmful traditional practices such as female genital mutilation and early marriage bring. It is also worse than the other harmful traditional practices as it poses virgin pregnancy as an additional consequence. Therefore, it is suggested that international and national organizations working on issues related to harmful traditional practice should pay due attention to *Shilshalo* just like what they are doing with female genital mutilation and early marriage.

**Electronic supplementary material:**

The online version of this article (10.1186/s12914-018-0179-x) contains supplementary material, which is available to authorized users.

## Background

All social groups in the world have specific practices and beliefs which often have strong cultural underpinnings. These can be either positive or negative. Almost all societies have positive cultural practices that are beneficial to all members. But, there are also practices which are harmful to specific groups, for example, for women and children. These harmful cultural practices are often dubbed as Harmful Traditional Practices (HTPs) [1].

It is a mere fact that HTPs are global problems affecting millions of women and girls of all culture, religion, socio-economic strata, educational levels and other diversities [1]. HTPs pose grave consequences especially on women and girls who are subject to them. Mostly, these segments of the population face physical damage, psychological problems, health complications including sexually transmitted infection, social stigmatization, denial of educational and other opportunities, inability to control their reproductive right and the like [2]. In precise terms, HTPs are used as a weapon to keep women and girls in subordinate positions by denying their health, social, economic and human rights [3].

Even though various international, regional and national legal instruments have been used to eliminate HTPs, every year millions of women and girls across the world are still the victims of different harmful traditional practices [4]. HTPs are plenty in number, but international and national agencies have given priority only to certain types of HTPs by considering their geographical coverage. Among the recognized HTPs, dowry, female infanticide, early marriage, female genital mutilation (FGM) and inheritance marriage are the major ones [3]. However, the authors of this article believe that geographical coverage should not be the only criterion to label HTPs as major or minor social problems. Rather, HTPs that are practiced by minority groups should also get equal attention by the international and national agencies working on issues related to HTPs.

Similarly, there are several harmful traditional practices in Ethiopia; nonetheless, the nation has given emphasis only to a limited number of HTPs by considering the geographical area covered by the HTPs. In this regard, Ethiopia has identified 12 major HTPs that need intervention. These are FGM, early marriage, uvula cutting, milk teeth extraction, marriage by abduction, food prohibition, work restriction, massaging of the abdomen of pregnant women, excessive feasts, incision, and inheritance marriage [5].

The Amhara National Regional state, the second largest state of Ethiopia, on its part identified FGM, early marriage, milk teeth extraction, and uvula cutting as the major HTPs that need special focus in the region [6]. However, *Shilshalo* is not yet recognized as a major HTP though it has been practiced in the region for many years. The reason why the region failed to treat it together with the other four HTPs is not because it has less adverse impact on the overall wellbeing of women; rather it is practiced only by minority groups of people, namely *Argoba* community. Given this fact, it is hardly possible to get information about the overall features of *Shilshalo*. Therefore, this study attempted to generate a deep empirical investigation about the practices, consequences and causes of *Shilshalo*.

## Methodology

### Description of the study area

*Argoba* is one of the ethnic groups found in Amhara National Regional State of Ethiopia. The *Argoba* Nationality special *Woreda* [an autonomous administrative region or district], was founded in June 2006. It is found in *South Wollo* Zone of Amhara National Regional State. It is bordered with *Kalu Woreda* of *South Wollo* zone in the north and northwest, *Dewa Chefa Woreda* of *Oromia* Zone in the south, and *Telalak Woreda* of Afar Regional State in the east. The center of the *Woreda* is *Medina*, a small town 85 Kms and 375Kms far from *Dessie* and Addis Ababa, respectively [7].

With an area of 30,918.70 Km^2^, the *Argoba* Nationality special *Woreda* consists of seven rural *kebeles* [the lowest administrative region under *Woreda*]. It is a home for about 40,000 people (22,715 Male; 17,594 Female). The community is entirely Islam. Only some of the government employers, who came from other areas, are non-Islam. With respect to the economic activity, 99.5% of the total population is engaged in agricultural activities. Only the rest 0.5% participate in trade and other service economy [7].

### Research approach

Qualitative approach was employed in this study. Among the different kinds of qualitative research designs, case study design was used since the nature of the study calls for an empirical investigation of a particular phenomenon within its real life context. Hence, exploratory case study design was used to investigate the features of *Shilshalo* by taking the setting and context of *Argoba* community into account.

### Data gathering instruments and data collection procedures

The information about *Shilshalo* was elicited from the participants of this study through in-depth interview, key informant interview, and focus group discussion. To this effect, in-depth interviews were held with 8 girls and 6 boys and semi-structured interviews were conducted with 8 key informants comprised of religious leaders, elders, and health professionals. The in-depth and semi-structure interview guidelines were developed and used only for this study purpose (See Additional file 1).

Since *Shilshalo* is practiced by boys and girls in the stage of puberty before marriage, the boys and girls were purposively selected based on two inclusion criteria: having the experience of *Shilshalo* and being in the age range of 15 and 18. Before interviews were held with boys and girls, written consent from their parents and oral assent from them were obtained. Each interview lasted for an hour on average. The in-depth interviews were held in Amharic. The interview focused on issues such as why and how the teenagers practiced *Shilshalo* and the harms it brought to them.

Key informant interviews were held with two groups of purposively selected participants including two male religious leaders, two elderly women, two elderly men and two female health professionals. The first group consisted of religious leaders, and elderly women and men who could provide explanations about the socio-cultural rationales of performing the *Shilshalo*. The second group involved *Kebele* health professionals who could clarify the potential harm of practicing *Shilshalo* on the wellbeing of girls. A face-to-face interview was undertaken in Amharic with each of the key informants.

To validate the interview data and to get collective information, three separate focus group discussions were conducted with girls, boys and community members. Also, three discussion guidelines were prepared for each group of discussants to properly moderate the focus group discussion (See Additional file 2). Each focus group discussion consisted of eight individuals; and in total 24 people (12 women/girls and 12 men/boys) participated in the discussions. All focus group discussions were moderated by the researchers, and tape recording was used to capture the reflection of both interviewees and focus group discussants.

### Data analysis

The data were analyzed using qualitative thematic analysis technique, involving the following stages. First, the audio recorded data gained from interview and focus group discussions were transcribed. Then, the transcribed data were translated from Amharic to English. The English version of the raw data was read repeatedly to maintain familiarity (See Additional file 3). Next to this, the data were sorted and arranged into different types depending on the sources of the information. After that, detailed analysis was done with a coding process. This involved the process of organizing the material into chunks before bringing meaning to those chunks. And, it involved taking text data in categories and labeling those categories with themes. Finally, the data were interpreted and analyzed qualitatively.

### Trustworthiness of the study

Trustworthiness is seen as the strength of a research. It is used to determine whether the findings are accurate from the stand point of the researchers, the participants, or the readers of the account [8]. To ensure the trustworthiness of this qualitative study, we triangulated different data sources of information by examining evidence from the source and used it to build a coherent justification for themes. In addition to this, we used easy and simple language and description to convey the findings.

## Results

### The practice of *Shilshalo*

According to research participants, *Shilshalo* is a traditional sexual practice done by unmarried young boys and girls of the *Argoba* community. Boys and girls were playing a kind of sexual activity which is characterized with no actual insertion of the male’s genital organ (penis) into the female’s genital organ (vagina), but apart from this, other sexual activities are allowed in this sexual play. An old *Argoba* man explained this harmful traditional practice as follows.

*Shilshalo is a sexual play which a young unmarried girl and a young unmarried boy perform. A girl may start earlier than a boy, at 10 or 11 or 12 years age whereas the boy may start at the age of 14 years or later. They continue playing until she or he marries. Once they become friends, they play it when they get favorable conditions. When they are ready to play, they put their bodies in contact, especially making use of their hands. They also kiss each other, and finally the girl sleeps sticking her legs together, and the boy then brushes her thigh up to her vagina with his penis. He continues rubbing until he reaches at the state of orgasm and ejaculates his fluids there.*

From the above account, one can understand that the practice is done by teenagers though the starting age varies between boys and girls. The girls might begin the practice earlier than boys, which on average lies in the age range of 10 to 12 years, whereas boys usually begin at the age of 14 and above. The youngsters take this play as a means of getting sexual fantasy without considering the potential consequences that result from it. They use their body parts such as lips, hands, thighs and sexual organs to practice it. Unlike the common sexual intercourse, in *Shilshalo*, a boy ejaculates his sperm around the area he brushed, i.e. her thigh and areas around her vagina.

The results of this study showed that there are certain procedures followed in practicing *Shilshalo*. At the beginning, the boy is expected to express his love for a girl he loves. If she accepts his request, she expresses her agreement by putting jewelry on his neck or hand. Since then, they become sexual */Shilshalo/* partners so that they could practice everything that gives them sexual pleasure except practicing the actual sexual intercourse. This process was well articulated by the account of the following participant.

*I remember how I started playing Shilshalo. When I was 13 years old, one boy approached me and asked me to be his partner. As that time, I feared to give response, so he stayed for some time with no any answer from me. On day, while I was collecting firewood, he again asked me to be his partner. At that time, I expressed my agreement by putting a Musebeha (a jewelry made from a chain of iron and used as a gift by girls to express their willingness for boys’ love request) on his neck. After two years of joyful time, we separated when I married another man.*

This study also found out that a boy often allows his friends to enjoy with his girlfriend. This implies that the practice of *Shilshalo* allows a girl to play with more than one boy. In this regard, one of the participants shared her experience as follows:

*Playing Shilshalo with a lover is amusing but sometimes boys force girls to play it with their friends. In my two years stay with my boyfriend, I had encountered this problem for four times: once with a boy, once with two and twice with three of his friends. I used to enjoy when my boyfriend ejaculated his fluid on my body. However, when his friends ejaculated on my body I felt distasteful and it did not bring any joy for me. Although I disliked it too much, I did it for the sake of satisfying the interest of my boyfriend.*

The above narration shows that there is a hierarchal relationship between boys and girls during *Shilshalo*. The boy shows his masculine behavior by forcing his girlfriend to perform it with his friends. However, girls remain submissive and for that matter they do not have the power to oppose the interest of their boyfriends. Due to this reason, therefore, most girls do *Shilshalo* with many boys without their consent.

### Consequences of *Shilshalo*

The study found that *Shilshalo* brings devastating consequences upon the overall wellbeing of girls. The major problems are pointed out and discussed hereunder.

#### Virgin pregnancy

*Shilshalo* entertains the out flow of the male sperm on areas around the outer part of a girl’s vagina. That gives the possibility of a sperm cell entering the vagina as a result of which virgin pregnancy could occur. Thus, virgin pregnancy could be taken as a unique effect of *Shilshalo*. This painful pregnancy was witnessed by many health professionals who were working in *Argoba* district. For instance, a nurse shared what she observed as follows;

*I got a virgin pregnant girl when I was working at Chira Health Center in 2014. A girl who was 15 years old came to the health center with her mother. Since the girl feared to tell me what she faced, her mother began to explain what was happening on the health of her daughter. Unfortunately, all the cases she told me about her daughter could be taken as symptoms of pregnancy. Therefore, I ordered the girl to take a urine examination so as to check whether she was pregnant or not. However, her mother claimed that her daughter could never be pregnant. The mother told me that her daughter’s virginity was checked by a health professional. Sadly, the urine result was positive i.e. she was virgin but pregnant. I then asked the girl if she was involved Shilshalo or not. She confirmed that she had participated in the play. Then I told the test result for her mother and explained to her as it happened due to Shilshalo. The mother was shocked and asked me to do all possible options to terminate the fetus. However, I politely advised her not to take any action; otherwise she would lose her daughter.*

The narration of the above key informant reflected that *Shilshalo* as a sexual practice could become a cause for unintended pregnancy. The practice was done by brushing the girl’s genital organ by a male’s penis (without inserting the penis inside) and an outflow of a sperm cell on the exterior of the vagina. The sperm cell could then enter into the uterus via a tiny hole of virgin vagina. It is scientifically true that pregnancy might occur as long as the male sperm cell and the female cell coincide whether a female is virgin or not. That means virginity could not be a guarantee for protecting a sperm cell from entering into the uterus. It is, therefore, concluded that *Shilshalo* is a harmful traditional practice that exposes girls to experience virgin pregnancy.

#### Psycho-social consequence

Based on the findings of this study, *Shilshalo* brings psycho-social consequences upon girls especially on those who experience virgin pregnancy. A 16 years old virgin girl who was a victim of teenage pregnancy described her story as follows:

*It was better to be silent rather than think of what happened to me. It was better for me to die. I got pregnant without losing my virginity, but nobody including my parents believed me; they just thought I was lying. They took it as a joke and blamed me as I did open sexual intercourse during Shilshalo. The only option I had was to migrate outside Ethiopia. There are brokers who facilitate outmigration in a hidden way. Now, I am waiting for them to do something for me.*

As the experience of the above victim demonstrated, girls experiencing unintended pregnancy face stigma that comes from community members including parents. These girls were unable to cope with the societal stigma and consequently they develop multiple psychological problems such as low self-esteem, loneliness, anxiety, confusion, loss of hope, and frustration. Although the community perceives those virgin pregnant girls as if they committed open sexual intercourse, the actors responded that they were not doing open sexual intercourse rather they practice it in a very safe way since it brings societal punishment on them. This implies that *Shilshalo* has limited potential to expose adolescents for open sexual intercourse.

#### Physical harm

According to the participants of this study, teenager girls had also faced physical harm due to the practice of *Shilshalo*. These include body injuries, wound, and scratches by splinters, pricked by thorn and body fatigue. In line with this, a key informant woman said, “*in the course of playing, there could be body injuries or wound on both of us especially when we practiced it on unsafe areas. However, girls frequently experience injuries since they often sleep on the ground while boys on top.”*

Another woman also shared her experience by saying:

*I hated Shilshalo when I played it with my partner’s friends. His friends did not worry about me. They used excessive force and make me exhausted. In spite of this hardship, I never tried to refuse this practice fearing that my boyfriend would be annoyed if I failed to satisfy his friends’ needs.*

From the above reflection, we could recognize that girls were facing physical injuries while they practice *Shilshalo*. The injury occurs due to the nature of the practice as it lets girls to practice *Shilshalo* with more than one boy, sleeping on the ground.

#### Sexually transmitted infection (STI)

As it is discussed earlier, when boys and girls do *Shilshalo* they use their lips and sexual organs to gratify their sexual desires. This practice would in turn expose them to be infected with sexually transmitted infection including HIV/AIDS since there was high possibility of contamination of blood and sexual fluids. A key informant nurse at *Senkele* Health Center described this problem by saying:

*If there is bleeding or wound on their lips or areas around genital organs, STIs including HIV/AIDS can be transmitted from one person to the other. For girls, the possibility of being infected with such disease is so high since the practice is followed by the outflow of sperm cells at the gate of the vagina which may result in the inflow of sperm cells into the vagina. What I said is based on medical principle rather than by actually observing of the case as it is difficult to identify the case unlike the cases of pregnancy*.

From the above idea of the health worker, one could understand that the practice had the potential to transmit STIs from one to the other. This might be worse for a girl than a boy. A boy might be exposed to STIs only if there is a wound around his penis. Whereas a girl could be more vulnerable to such problems since a male semen could easily inflow into the vagina. Moreover, the responsibility of a girl to entertain many boys has worsened the problem further.

### Reasons to practice S*hilshalo*

According to the result of the study, most members of *Argoba* community had considered *shilshalo* as a beneficial cultural practice, only because it lets the teenagers to gratify their sexual feelings. They thought that it is useful to prepare young girls and boys for the expectation married life. That means they believed that the practice helps the youngsters to share love with their friends and at the same time to respect cultural criteria for marriage--a girl should stay virgin until marriage. It is true that in a patriarchal society, virginity of girls is associated with purity and honor but in contrast, virginity of boys is not as such an issue. Since the study community was a patriarchal community, they understood virginity in the sense of the above notion. However, the study communities, unlike the other patriarchal societies, did not believe that protecting girls from any sexual relationship with boys is not a long lasting option to keep girls’ virginity because love affiliation between youngsters is a natural phenomenon. By recognizing these facts, therefore, the *Argoba* community had created *shilshalo* as means to protect girls from losing of virginity before marriage. In this regard one participant said:

*In our culture, a girl must be virgin to be a wife of someone. Assume if a man married a girl with wedding ceremony and if he identified her as she is not virgin, he will immediately send her back to her families. And the girls’ families will consider this as something that degraded their dignity; and she will then be discriminated by the society as she violates the norm of the society. However, our culture does not want to control sexual behavior of girls though their virginity is not compromised. Therefore, shilshalo is created as a method to make girls to be able to preserve their virginity and at the same time to perform sexual activity.*

The other reason raised for the favor of *shilshalo* is mothers’ opinion on it. They took it as an indicator for the fate of their daughters. As to them, a girl who participate in the practice (has a boyfriend) is considered as beautiful and she will be wanted for married life. If not, it will be interpreted otherwise and she will remain unmarried. Therefore, the mothers used it to know if or not their daughters are wanted for marriage. In connection to this idea, one woman shared her experience of treating her daughters as:

*I have three daughters. I have always encouraged my daughters to practice shilshalo. I take it as a means of checking the fate of my daughters in terms of accessing husbands in the near future. If my daughters do not have boyfriends, I thought they will not have a married life in the future. But if they have boyfriends, it implies that they will be wanted by males for marriage.*

From the above explanation one can conclude that the mothers were those who favored *shilshalo.* They perceived their daughters engagement in the practice of *shilshalo* as a guarantee for their future married life. They assumed a girl participates in *shilshalo,* mean, she is a one wanted for marriage, otherwise she is considered as unfit for married life. Likewise, the information collected from the focus group discussants showed that although the mothers stand in support of the practice of *shilshalo*, it was difficult to say men had no role in sustaining the practice. While mothers used the practice as a means of assuring their daughters’ destiny regarding married life, the fathers did not take any action on it. This indicates that the fathers had favorable attitude towards it since they did not restrict their sons and daughters to involve in the *shilshalo*practice.

The study also found out that *Argoba* girls had a positive attitude towards *shilshalo*. In the *Argoba* culture, a girl was expected to marry a person whom her families choose. This implies that she did not have a right to select whom she wanted to marry. Therefore, girls had taken ‘*shilshalo*as an opportunity to entertain love relationship with the one they might not get in their life. One young girl clearly explained this idea by saying:

*Love coupled with adolescence feeling initiated me to play shilshalo. In our culture, being a female mean, you do not have a right upon choosing your future life partner. Our families have decided all about us. As a human being we look something that could bring about joy. For example, in my case I want to marry the man I loved but my culture does not allow me to do that. So I used shilshalo to get my beloved one before I married someone that I may not like. However, if I make an open sexual intercourse by mistake, I would lose my virginity and this would in turn create different societal problems upon me. Therefore, in order to escape from such bad condition I take care when I perform it.*

The *Argoba* boys had also supported the practice of *shilshalo* but their justification is a little bit different from what the girls mentioned. In the *Argoba* culture, there were different expectations for a boy and a girl to engage in marriage life. The boy should be self-reliance for basic needs. Whereas a girl was expected to marry someone her families prefer. However, most of the boys found out fulfilling household materials, which are basic to live with his future wife, was a time consuming activity. Boys did not want to wait their wedding day to have sexual relationship with girls; and because of this reason they had practiced *shilshalo* before marriage. One of the interviewed boys strengthened this view as:

*To marry a girl, I should first secure the basic goods. Once, I secured it, I can marry a girl I loved. But getting enough resource for life is a big problem for me. So that, I have used shilshalo as a way of getting sexual fantasy until I become capable enough to marry someone*.

The above case stories showed that boys and girls had strong zeal to practice *‘shilshalo’* at their adolescent age. They explained that the practice helped them to share love with their partner before they marry someone. Especially, girls considered it as the only means to enjoy with someone she loved before marrying some other person chosen by her families. Similarly, a boy perceived it as an opportunity to satisfy his sexual desires until he became financially capable to marry a girl. Both boys and girls did not expect any harm from it as long as it was properly performed. They perceived that the harm resulted from the practice occurred due to improper practice. Otherwise, it was safe as they thought.

However, the religious leaders had openly opposed the practice. Most of *Argoba* community members were follower of Islamic religion and their religious leaders had often taught the community not to practice *shilshalo*. One of the religious leaders explained this matter by saying:

*Shilshalo has been practiced by our community for ages. Our religion forbids any form of sexual practice before marriage. Even any form of relationship between young boys and girls before marriage is considered as a sin. We (religious leaders) have often preached this to our followers but most of the community members are reluctant to behave as per our teaching.*

From the standpoint of the above religious leader it was possible to say that the practice does not have religious grounds. The Islam religion prohibited any form of sexual practice before marriage. In contrast to this religious teaching, the *Argoba* community has still practiced it.

## Discussion

Many of the medical, psychological and epidemiological researches clearly assert that sexual experience is assumed to be inappropriate for adolescents and preserved for adults [9]. As opposed to this idea, the *Argoba* community promotes the early sexual experience of young boys and girls by adopting *shilshalo* which is an uncommon type of harmful traditional practice in the rest of the world. *Shilshalo is* performed by practicing all sorts of sexual activities with the exception of inserting the male genital organ (penis) into the female sexual organ (vagina). This feature of *Shilshalo* makes it different from masturbation since *Shilshalo* is a kind of sexual stimulation done between partners while masturbation is to give oneself a sexual pleasure by rubbing one’s own sexual organ.

Most harmful traditional practices including FGM and early marriage have been practiced against girls with forceful action meaning that the will of girls is not respected [5]. Unlike these harmful traditional practices, *Shilshalo* is not practiced by forcing girls to perform it rather the option is given for them although it has negative effect on their lives. However, this does not mean that there was no family pressure on the practice. As the present study showed, the mothers were the one who encouraged their daughter to engage in *Shilshalo*.

Some studies conducted in Ethiopia stated that the practice of abduction is often accompanied with physical injury since the abductor uses force [9, 10]. Similarly, girls practicing *Shilshalo* face physical injury especially when they do it with many boys. Most boys who perform *Shilshalo* with their friends’ partner would use excessive physical force and make the girls to be weakened.

In some African countries, women aged 15–24 are being infected with HIV/AIDS at rates five times greater than men with the same age category. This higher rate can be described by the fact that girls are more susceptible to contracting HIV because they are married to older men due to the presence of early marriage practice [11, 12]. This indicates that early marriage has negative health impact on young girls as it makes them vulnerable to HIV infection. Also, predisposes girls to HIV infection. The studies conducted in sub-Saharan countries revealed that the same equipment was used on 15–20 girls to mutilate their genital organs in a traditional way. And, this circumstance would, in turn, expose girls to be infected with HIV [13–15]. Similarly, the present study postulated that *Shilshalo* might play a role in the transmission of HIV/AIDS. It is practiced in a way that the boy ejaculates on the gate of the girl’s vagina and the sperm cell then possibly flows into the inner part of the vagina and as a result of this, it gives chance for STIs including HIV/AIDS to be transmitted from one to the other especially from males to females. Apart from the STIs and related consequences, *Shilshalo* had also brought virgin pregnancy upon young girls. This virgin pregnancy was the very unique feature of *Shilshalo* and it is hard to find an HTP that has such kind of consequence all over the world. Besides, once girls experienced virgin pregnancy, they were excluded by the community and they would never marry someone else since virginity is the major prerequisite for marriage in the *Argoba* culture.

Almost all HTPs are done for some cultural-based reasons. For instance, the strongest reason for early marriage in Ethiopia is the desire to maintain the family’s prestige and popularity. It is also practiced by some parents just to create an alliance between two families for social, political and economic reasons [6]. When we see the reason associated with the practice of FGM in Ethiopia, it is practiced for making woman to able to do proper sexual intercourse and protecting her from prostitution [6]. Regarding rationales that underpin *Shishalo* to become long lasting traditional practice, there were different reasons justified by the community. To mention the major ones, it was believed to be a way of maintaining girls’ virginity. The mothers were also used it to cross check whether their daughter would get a husband or not in the future. When their daughters had boyfriend to practice *Shilshalo*, they would assume as if their daughters would surely get husbands. However, unlike the other HTPs, the victimized groups (girls and boys) were favoring the practice of *Shilshalo* because they considered it as an opportunity to entertain their sexual needs before marriage.

As a matter of fact, religion has strong association with HTPs especially with polygamy. The Islamic religious teachings encourage men to marry up to 4 women. In this case, religion has a direct connection with HTPs. However, the other canon of Islamic religion is that any sexual practice before marriage is forbidden [16]. Since *Shilshalo* is practiced before marriage, it did not get religious support rather it has been opposed by Islamic religious leaders. Given this wisdom, we can understand that religion is not always in favor of all forms of HTPs rather it depends on the nature of the HTPs.

## Conclusion

Based on the findings of this study, it was concluded that *Shilshalo* is basically practiced in the *Argoba* community to preserve young girls’ virginity until marriage. The community believes that restricting young girls from any sexual relationship is not a long lasting option to protect them from losing of their virginity before marriage, rather the community allows them to involve in a kind of sexual practice but without actual genital intercourse. While practicing *Shilshalo*, there is high possibility that the male’s sperm enters into the vagina. As a result, there are many girls who experience virgin pregnancy and they are also exposed to sexually transmitted diseases including HIV/AIDS. Once they experience virgin pregnancy, they are excluded by the society and their fate to get a husband would be in danger. Although *Shilshalo* has a devastating effect on the health and overall wellbeing of young girls, it has been still considered by most of the *Arogoba* people including the actors as a beneficial cultural practice. Hence, it was suggested that concerned bodies should take measures to eliminate this harmful traditional practice.

## Additional files

Additional file 1: In-depth and semi-structure interview guidelines used in doing conversation with interviewees and key informants. (DOCX 21 kb)

Additional file 2: Guidelines used in conducting discussions with focus group discussants. (DOCX 18 kb)

Additional file 3: Raw data used in the analysis. (DOCX 39 kb)

## Abbreviations

FGM: Female Genital Mutilation
HIV: Human Immunodeficiency Virus
HTP: Harmful Traditional Practice
SRH: Sexual and Reproductive Health
STI: Sexual Transmitted Infection
