# Supplementary material for: A harmful traditional practice exposing young girls to experience virgin pregnancy (Shilshalo): a qualitative study in Argoba community, Amhara National Regional State, Ethiopia
Source: BMC Int Health Hum Rights. 2018 Nov 20;18:42. doi: 10.1186/s12914-018-0179-x (PMC6247749; doi:10.1186/s12914-018-0179-x)
Supplement: Supplementary file 1 — In-depth and semi-structure interview guidelines used in doing conversation with interviewees and key informants. (DOCX 21 kb) [file 12914_2018_179_MOESM1_ESM.docx]

**I. Interview Guides**

**1.1 In-depth interview guideline for girls and boys**

**Introduction**

This study is being conducted to gather data on the ‘Practice, causes and consequences of *Shilshalo*’ in *Argoba* community’. The study is aimed to generate data that will be used for academic and future intervention purposes only. The successful accomplishment of this study is highly determined by the data obtained from you. The information that you give will be kept strictly confidential.

Thank you!

1. **Personal questions**
2. Would you tell me your name?
3. Would you tell me your age?
4. Would you tell me about your marital status?
5. Would you tell me about your level education?
6. **Questions related to ‘*Shilshalo*’**
7. How do you look ‘*Shilshal*o’? Is that bad or good cultural practice for you?
8. How and when ‘*Shilshal*o’ is practiced in your community?
9. What is underline causes for practicing ‘*Shilshal*o’ in your community?
10. Have you ever participated in ‘*Shilshal*o’ practice?
11. If your response is yes for question number 8, tell me how you engage in the practice and brief the activities that you undertake in performing it?
12. What kind of consequences ‘*Shilshal*o’ has brought up on you or on other actors?

**Probing points**

- Health consequences
- Social consequences
- Psychological consequences

1. Do you have anything to say more?

Thank you for your cooperation!

**1.2 Semi-structure interview guideline for elders**

**Introduction**

This study is being conducted to gather data on the ‘Practice, causes and consequences of *Shilshalo*’ in *Argoba* community’. The study is aimed to generate data that will be used for academic and future intervention purposes only. The successful accomplishment of this study is highly determined by the data obtained from you. The information that you give will be kept strictly confidential.

Thank you!

1. **Personal questions**
2. Would you tell me your name?
3. Would you tell me your age?
4. Would you tell me about your marital status?
5. Would you tell me about your level education?
6. Would you tell me about your work?
7. Tell me about your family condition?
8. **Questions related to ‘*Shilshalo*’**
9. How do you look ‘*Shilshal*o’? Is that bad or good cultural practice for you?
10. How and when ‘*Shilshal*o’ is practiced in your community?
11. What is underline causes for practicing ‘*Shilshal*o’ in your community?
12. What kind of consequences ‘*Shilshal*o’ has brought up on actors?

**Probing points**

- Health consequences
- Social consequences
- Psychological consequences

1. Do you have anything to say more?

Thank you for your cooperation!

**1.3 Semi-structure interview guideline for religious leaders**

**Introduction**

This study is being conducted to gather data on the ‘Practice, causes and consequences of *Shilshalo*’ in *Argoba* community’. The study is aimed to generate data that will be used for academic and future intervention purposes only. The successful accomplishment of this study is highly determined by the data obtained from you. The information that you give will be kept strictly confidential.

Thank you!

1. **Personal questions**
2. Would you tell me your name?
3. Would you tell me your age?
4. Would you tell me about your marital status?
5. Would you tell me about your level education?
6. Would you tell me about your religious role?
7. **Questions related to ‘*Shilshalo*’**
8. How do you look ‘*Shilshal*o’? Is that bad or good cultural practice for you?
9. How and when ‘*Shilshal*o’ is practiced in your community?
10. What is underline causes for practicing ‘*Shilshal*o’ in your community?
11. Does ‘*Shilshal*o’ have a religious ground? Explain it in brief?
12. What kind of consequences ‘*Shilshal*o’ has brought up on actors?

**Probing points**

- Health consequences
- Social consequences
- Psychological consequences

1. Do you have anything to say more?

Thank you for your cooperation!

**1.3 Semi-structure interview guideline for health extension workers**

**Introduction**

This study is being conducted to gather data on the ‘Practice, causes and consequences of *Shilshalo*’ in *Argoba* community’. The study is aimed to generate data that will be used for academic and future intervention purposes only. The successful accomplishment of this study is highly determined by the data obtained from you. The information that you give will be kept strictly confidential.

Thank you!

1. **Personal questions**
2. Would you tell me your name?
3. Would you tell me your age?
4. Would you tell me about your marital status?
5. Would you tell me about your level education?
6. Would you tell me about your working status?
7. For how many years you are working in the study area as health worker?
8. **Questions related to ‘*Shilshalo*’**
9. What kind of information you have about ‘*Shilshal*o’?
10. How do you look ‘*Shilshal*o’? Is that bad or good cultural practice for you?
11. What is underline causes for practicing ‘*Shilshal*o’ in your community?
12. What kind of consequences ‘*Shilshal*o’ has brought up on actors? Share us what you have heard or observed as a health worker?

**Probing points**

- Health consequences
- Social consequences
- Psychological consequences

1. Do you have anything to say more?

Thank you for your cooperation!
