# Supplementary material for: A harmful traditional practice exposing young girls to experience virgin pregnancy (Shilshalo): a qualitative study in Argoba community, Amhara National Regional State, Ethiopia
Source: BMC Int Health Hum Rights. 2018 Nov 20;18:42. doi: 10.1186/s12914-018-0179-x (PMC6247749; doi:10.1186/s12914-018-0179-x)
Supplement: Supplementary file 2 — Guidelines used in conducting discussions with focus group discussants. (DOCX 18 kb) [file 12914_2018_179_MOESM2_ESM.docx]

**I. FGD Guides**

**1.1 Focus group discussion guideline for girls**

**Introduction**

This study is being conducted to gather data on the ‘Practice, causes and consequences of *Shilshalo*’ in *Argoba* community’. The study is aimed to generate data that will be used for academic and future intervention purposes only. The successful accomplishment of this study is highly determined by the data obtained from you. The information that you give will be kept strictly confidential.

Thank you!

1. **Personal questions**
2. Would you tell us your name?
3. Would you tell us about your level education?
4. **Questions related to ‘*Shilshalo*’**
5. Discuss about the practice of ‘*Shilshal*o’.
6. Discuss about the underline causes for practicing ‘*Shilshal*o’ in your community.
7. Discuss about the motivating factors that push girls to engage in the practice.
8. Discuss about the consequences that resulted from ‘*Shilshal*o’.

**Probing points**

- Health consequences
- Social consequences
- Psychological consequences

1. Do you have anything to add?

Thank you for your cooperation!

**1.2 Focus group discussion guideline for boys**

**Introduction**

This study is being conducted to gather data on the ‘Practice, causes and consequences of *Shilshalo*’ in *Argoba* community’. The study is aimed to generate data that will be used for academic and future intervention purposes only. The successful accomplishment of this study is highly determined by the data obtained from you. The information that you give will be kept strictly confidential.

Thank you!

1. **Personal questions**
2. Would you tell us your name?
3. Would you tell us about your level education?
4. **Questions related to ‘*Shilshalo*’**
5. Discuss about the practice of ‘*Shilshal*o’.
6. Discuss about the underline causes for practicing ‘*Shilshal*o’ in your community.
7. Discuss about the motivating factors that push boys to engage in the practice.
8. Discuss about the consequences that resulted from ‘*Shilshal*o’.

**Probing points**

- Health consequences
- Social consequences
- Psychological consequences

1. Do you have anything to add?

Thank you for your cooperation!

**1.3 Focus group discussion guideline for community members**

**Introduction**

This study is being conducted to gather data on the ‘Practice, causes and consequences of *Shilshalo*’ in *Argoba* community’. The study is aimed to generate data that will be used for academic and future intervention purposes only. The successful accomplishment of this study is highly determined by the data obtained from you. The information that you give will be kept strictly confidential.

Thank you!

1. **Personal questions**
2. Would you tell us your name?
3. Would you tell us about your marital status?
4. Would you tell us about your work?
5. Please tell us the numbers of sons and daughters you have?
6. **Questions related to ‘*Shilshalo*’**
7. Discuss about the practice of ‘*Shilshal*o’.
8. Discuss about the underline causes for practicing ‘*Shilshal*o’ in your community.
9. Discuss about the consequences that resulted from ‘*Shilshal*o’.

**Probing points**

- Health consequences
- Social consequences
- Psychological consequences

1. Do you have anything to add?

Thank you for your cooperation!
