# Supplementary material for: A harmful traditional practice exposing young girls to experience virgin pregnancy (Shilshalo): a qualitative study in Argoba community, Amhara National Regional State, Ethiopia
Source: BMC Int Health Hum Rights. 2018 Nov 20;18:42. doi: 10.1186/s12914-018-0179-x (PMC6247749; doi:10.1186/s12914-018-0179-x)
Supplement: Supplementary file 3 — Raw data used in the analysis. (DOCX 39 kb) [file 12914_2018_179_MOESM3_ESM.docx]

**Raw Data for study entitled: ‘A Harmful Traditional Practice Exposing Young Girls to Experience Virgin Pregnancy (*Shilshalo*): A Qualitative Study in Argoba Community, Amhara Regional State, Ethiopia’.**

Participant 1

*‘Shilshalo’ is a sexual play performed by young unmarried girl and boy. A girl may start earlier than a boy, at 10 or 11 or 12 years age whereas the boy may start at the age of 14 years or later. They continue playing until she or he marries. Once they become a friend, they play it when they get favorable condition. When they are ready to play, they touch their bodies to each other with their hands, they also kiss each other and finally the girl sleeps by sticking her legs together and the boy then brushes her thigh up to her vagina with his penis. He continues rubbing until he reaches at the state of orgasm and ejaculate his fluids there.*

*…even if it looks like a one to one relation, sometimes there may be more than one boy play with a single girl. This case may happen if a boy lets his peer friends to play with his girlfriend. When this occurs the girl is expected to do like she did with her boyfriend. However, they go sometimes far from what is expected either due to being carelessness or geared by their sexual feelings. In this case, especially a girl may face problems like losing of virginity or unwanted pregnancy which are not accepted by the community and her family too.*

Participant 2

*I remember how I started playing Shilshalo. When I was 13 years old, one boy approached me and asked me to be his partner. As that time, I feared to give response, so he stayed for some time with no any answer from me. On day, while I was collecting firewood, he again asked me to be his partner. At that time, I expressed my agreement by putting a Musebeha (a jewelry made from a chain of iron and used as a gift by girls to express their willingness for boys’ love request) on his neck. After two years of joyful time, we separated when I married another man.*

*That day, I felt something which was unusual to me, but it gave me a pleasure. When we returned back to home, my peer friends talked to each other, but I couldn’t hear what they talked. I pondered only a pleasure I had got. Then on wards, I longed for the day my boyfriend met me and play ‘shilshalo’ to test the pleasure of the playing what I heard ever in my life. Three or four days later after our first body contact, I together with my peer friends went to the forest to collect fire wood , but nothing new happened other than our first day experience. After that day, my love increased. Everything I sang, hummed etc. were his name when I worked in the home.*

*One day, after a long yearn, one of my peer friends came in my home and told me as he waited me in her home and her mother went to in the market place. When she told me I did not know what I felt. Something invaded my body. I became very happy. We went to my friend’s home and I got him there as awaited me. He brought perfume and soap. Then, we touched and kissed each other. Later on he wore out his ‘gildim’ (a skirt worn by a male), I slept, and then (she bowed and laughed), what did I say (She laughed), from my thigh to my-------(she feared to mention a vagina), he kneaded , after he moistened around my genital organ, we have finished. After that he opened a door and went out of the home. As soon as he got out, my friend came and I went to my home after we played a couple of minutes with my peer friend.*

*After that day, he became the only thought what I did. Always I thought, getting him and playing with him. He also brought soap, perfume and sweat when he went to market place. After that our loves become increased. When I went to everywhere with my peers and saw him, I went far away from my friends and we (me & he) got each other freely. After that we did what we yearned for (she laughed). Sometimes, if there was a wedding or other banquet event, we participated in the song and eventually enjoyed our pleasure by going to hidden area.*

*Playing Shilshalo with a lover is amusing but sometimes boys force girls to play it with their friends. In my two years stay with my boyfriend, I had encountered this problem for four times: once with a boy, once with two and twice with three of his friends. I used to enjoy when my boyfriend ejaculated his fluid on my body. However, when his friends ejaculated on my body I felt distasteful and it did not bring any joy for me. Although I disliked it too much, I did it for the sake of satisfying the interest of my boyfriend. After two years and four or five month joyful stay, I engaged in marriage. He gave me umbrella, box, perfume and soap in my wedding day as expected.*

Participant 3

*I was around 15 years old when I started shilshalo. In our village, there was a girl who I loved. One day I went to her where she watched and protected sorghum from u attack at agricultural field. I requested her to be my girlfriend, but she replied that “she is yet a kid, so she does not want things like this”. Although she said like that, I waited her everywhere: at the road along streams, at forest area of collecting fire wood and had importunate her. At one night in the song of a wedding, the song and dance become warmed and those who had friend coupled and played everywhere under the fence and backyards. As that time a girl I had asked was in a role of leading the song and she praised my name in her song. I couldn’t believe. How it could be? I felt happy. Just she finished her leading role. I showed her a sign, so she came and gave me ‘musebeha’ by taking out from her neck. This means, I am ok for your request. Therefore, I become very happy. Then we went to a few distances from the center of the song and sat under a fence. After that we touched our body and kissed each other, but she rejected my interest of playing ‘shilshalo’. We made an appointment for the next day. In the coming day she went alone to fetch water. As a result, we went far away from the area of a stream and sat under one tree, we touched and kissed each other, when I wore my ‘gildim’, she slept, then I uncovered her dress and I did it (he laughed), up to I ejaculated there.*

*After that when my sexual feeling stretched me, I found her by going around her home until she saw me because once she saw me, she created a pretext to get out and then we enjoyed shilshalo.*

*We had lived for nearly two years with my first girlfriend. After this she had married. As a result, I made friendship with another girl. She also married in the next year. After this, I have stopped having a girlfriend because I learned that having a girlfriend mean subjected to a great cost for gifts.*

Participant 4

*Sometimes my friends might come in when I had an appointment with my girlfriend, they asked me to let them to play with her. I did not have any option. Therefore, I allowed them to play with her after I did. If they were more than one, they would do it turn by turn in the way I do on her.*

Participant 5

*....on the other day, I might face similar situation. As that time especially if I became on feeling that puffed up, I would face a challenge, so if I did not allow sharing my friend, who could give me his friend*?

Participant 6

*She did not say no, if she was not willing to entertain my friends, how she could be considered as my girlfriend. If she did like that, I left her and I would find another girl to be my friend.*

.

Participant 7

*Young boys and young girls play it everywhere as they consider the place hide them from others sight. They do not have other criterion to select it. They play it at wood/forest area, along the road when they go to fetch water, at their friend home as the time their families are not stay at their home.*

Participant 8

A*s long as it could hide us from the sight of other persons we played everywhere. Simply we played it by going far away from our peer friends when we collected fire wood. We did it in the sorghum when we watched it from bird, at my friends’ home if their families went away far from the village. We also experienced it at the backyard especially at night time. So, we selected the place only on the basis of its hiding situation rather than considering whether it is comfortable or not for sleeping. Simply we saw its upper part for the presence of harmful things like thorn, cutting stone etc. Otherwise if there is harm from such things we observed it after we shared out joy.*

Participant 1

*We have different religious festivals and socio- cultural events that bring an opportunity for the young boys and girls to play ‘shilshalo’. They need only something that made them come together. Although our religion prohibits such actions, the young use EID and Arefa as a good opportunity to collect at one center. At that moment they sing and dance at the night. Following this they become coupled and go far away from the center of the playing ground. Then they enjoy what they want to do with their beloved. It also brings a chance for those who have not a friend. It is a good opportunity to find a new friend.*

*The other events which collect youngsters together for singing and dancing are those related with wedding and giving birth. In our culture, wedding is escorted by other events which have a banquet. If there is a banquet mean there will be song and dance for the youngsters. Such wedding related events included ‘kibekebi’, ‘lefijele’ and wedding itself. In these all situations banquet is prepared. That means there will be eating, singing, and dancing, so the youngsters have got a chance to play with their friends, if they have a friend. If not they will find a one who fit their interests.*

*The other socio-culture event is the one related with giving birth. This is known as ‘melhotaye’, which has also a banquet and song. After eating, song and dance will continue at the night. The youngsters praise their friends by song. Then, they will do what they want from their friend, if they had a friend. Otherwise it is also a good opportunity to find a friend.*

Participant 11

*There were events that I waited for their occurrence. Among these ED and Arefa were those related with our religion. In these festivals we came together and played a traditional song and dance. In these programs, we (me and my girlfriend) praised each other when we got a chance of leading a song. Then, we went far away from a song place to a place where hidden us from anyone observation and made joy by kissing and doing ‘shilshalo’.*

*The other events which I impressed were those related with wedding and birth ceremony. That included ‘kibekebi’, lafijele’ and wedding itself. All these events had traditional song and dance ceremonies. In this case we sang and danced and we praised by singing. Then, we turned from the sight of our colleagues in order to get a pleasure. After that we returned back to the song. Another important event was related with a birth ceremony which is known as ‘melehotaye’ which had a banquet, song and dance ceremonies. We liked it because it created comfortable situation like that of wedding ceremonies, so these festivals and events were those we always looked forward to appear frequently.*

Participant 12

*There were social events and religious rituals that gave us a chance of coming together. In such conditions we came from different villages to one center. Then, we sang and danced, and then ‘zemed kezemdu ahya kamedu’. It means in English relative with relative and donkey with ash. Meaning a one who has a friend played with him/her and a one who had not a friend remained alone. Therefore, I enjoyed with my boyfriend in the name of these events and religious rituals.*

*These social events were related with wedding and giving birth. These events like kibekebi’,‘lefijele’ and wedding were those mentioned with wedding and ‘melehotaye’ with birth giving ceremony .Among the religious rituals ED and Arefa were those given us an opportunity of collecting in one center , so these were events and festivals that I became curious for their coming on.*

Participant 5

*Regar*ding *‘Shilshalo’ no convention or deal made by the community at all. Whether it should practice or not, or how it should transfer to the next generation. However, even when we consider from our time, one who knows about it told for those who did not know. That means the elder shared their experience to the younger. But on the contrary, no formal ways of transferring ‘Shilshalo’ from generation to generation has employed.*

Participant 13

*I did not remember specifically when and how I got information. But, when we were a child, yet not reached to play ‘Shilshalo’, we heard about it from our elder and eager to taste what our elder had talked to us. They had talked what playing of ‘Shilshalo’ with liked girl was enjoyable and amusing. In addition there were sayings that served as a means to make ‘Shilshalo’. These include: if a young boy did not play ‘Shilshalo’, how it could be possible to say him an adolescence, A young girl who did not play ‘Shilshalo’ mean, she would marry without testing love, so sayings like these urge us to be eager about it. As we heard, we (peer groups) talked each other about what we heard from our elders and became eager to test its pleasure. We had longed for the age that we are going to start playing ‘Shilshalo’. So, we had grown in this way and eventually we had reached and tasted its joy. Nevertheless, we heard a common bad talk too. The bad talk we heard was related with divergent of girls and the compensation provided by a male for their deflowering of a girl virgin.*

Participant 14

*I did not remember from whom, when and how I got information about ‘Shilshalo’ for the first time because it was told by everybody. No special occasion was conducted for the sake of teaching it, but we heard about it from our elders, when the mothers talked to each other at the coffee ceremony. After having this I shared with my peers what I heard from various source, so I grew by doing so. I became curious to apply the practice and looked forward for my age appropriate for the practice. When I reached to the age of the practice my mother informed me regarding the care what I should take. Without the guidance given by my mother, no one gave me an advice or regulation or knowledge about ‘Shilshalo’.*

Participant 15

*In our culture, a girl must be virgin to be a wife of someone. Assume if a man married a girl with wedding ceremony and if he identified her as she is not virgin, he will immediately return her back to her families. The girl’s family considers such act as something that degraded the honor of the family; and she will be then discriminated by the society since she violates the norm of the society. However, our culture does not want to control sexual behavior of girls though their virginity is uncompromised issue. Therefore, ‘‘Shilshalo’ is created as a method to make girls to be able to preserve their virginity and at the same time to perform sexual activity.*

*Regarding the way concluding their marriage, it is mostly the role of their families. The criterion for the boy to marry is fulfilling his home household materials that are basic to live with his wife. After fulfilling these boy can ask his family to engage a girl he liked. Then, his family can request her family through go between. Thus, a boy marrying process ends like this. In the case of a girl it is differ from that of a boy. In this case, a girl is expected to marry a person whom her families choose to be her husband. Rather, she does not have a right to select whom she wants to marry. So, she must accept what her families permit to do whether she likes or not.*

Participant 16

*To marry a girl, I should first secure the basic goods. Once, I secured it, I can marry a girl I liked. But getting enough resource for life is a big problem for me. So that, I have used ‘Shilshalo’ as a way out to enjoy myself until I become capable enough to marry someone*.

Participant 17

*There may be some members of our community other than those participate on the playing who favors the practice. As rumors showed the mothers are in favor of the playing. They take it as a means of checking the fate of their daughters whether she will be wanted or not for married life. A mother feels her daughter as ignorant, if she does not have a friend. When a girl does not get a ‘shilshalo’ friend, her mother think as her daughter will not have a married life for the future. She cannot be wanted by male. She may be remained a maid. So, I think the mothers used ‘Shilshalo’ as to escape from these fears in which the daughter may face. For this reason the mother may urge the daughters to participate in the practice, i.e. ‘Shilshalo’.*

*Regarding the young who participate in the game, I think they may motivate due to lack of their endurance to win over their adolescence feelings. I think as long as they face problems of overcoming adolescent feelings, the youngsters take it as a solution. They consider it as the only options to harmonize their problems with their culture.*

*I think they see it as an opportunity to play with the one he/she loves. For the boy it means the one that give him a chance to play with a girl he loves before she marry another. For the girl also it provides similar opportunity by keeping her virgin, So I think the young are initiated to practice ‘Shilshalo’ for these reasons.*

*With regard to objection against ‘Shilshalo’, the religious leaders could be taken as the only opposition. Our religious leaders preached ‘Shilshalo’ as one of ‘haram’ practice.*

*Regarding the male house headed approach to ‘Shilshalo’ it is difficult to determine whether they favored or opposed it. However, there have been sayings that fathers do not know the participation of their sons and daughters in ‘Shilshalo’. In my opinion which is not seem true. Imagine, the husband themselves had grown through this culture mean they knew what the young do, so it is a truth we lived.*

Participant 18

*In the past there was a thought that a beauty of one girl was measured by a number of young boys who asked her for friendship. That means this determine her fate of being wanted or not for married life. Well then, no mother needs her daughter remain unmarried thus she (the mother) encouraged her daughter to be agile so as to have a boyfriend. After once a daughter got a boyfriend a mother create conducive situation for her to play with her boyfriend like let alone her to go to collect fire wood, fetch water when she requests as well as leaving home when daughter’s boyfriend came at home. The mother role is mentoring a daughter to know the care she has to be taken in playing ‘Shilshalo’ rather than advising them not to practice it.*

*I have three daughters. I have always encouraged my daughters to practice ‘Shilshalo’. I take it as a means of checking the fate of my daughters in terms of accessing husbands in the near future. If my daughters do not have boyfriends, I thought they will not have a married life in the future. But if they have boyfriends, it implies that they will be wanted by males for marriage.*

Participant 19

*I heard as ‘Shilshalo’ has practiced in our community. Our religion forbids any form of sexual practice before marriage even any form of play between young boys and girls including any forms of celebration. We (religious leaders) have preached this to our followers though most of them have not changed it to practice.*

*Even we condemned the practice and taught men and to some extent women, we have played limited role to address the issues for young boys and young girls who are the actors of the practice*.

Participant 20

*People play it are those who are at the age of adolescent, they are on their fire age which drive them to give priority to their sexual feelings, but, they have not an opportunity to undertake sex. Instead to get a relief ‘Shilshalo provide an opportunity to carry on sexual needs , so the role it plays to cool down their sexual feelings at their fire age can be seen as an advantage*.

*Its benefits to the adolescents are unquestionable. Its participants are the unmarried young boys and young girls who have not any options to treat their sexual feelings. Therefore, there is no an interesting practices for them other than ‘Shilshalo’ to relax themselves*.

Participant 21

*There may be a harm/problem if they played it not properly. If they play it safely any problem cannot be occurred but if not they may have a sex instead of ‘Shilshalo’. If so she may lost her virgin and may conceive a child. This brings social problem on a girl. A problem on the boy may be raised if a girl excelled a name of a boy when her husband hit her for the absence of her virgin. Therefore, a boy may be expected to give compensation to the husband of his ex-girlfriend for his deflowering of a virgin. But if her husband preferred a conflict to a compensation he expected to received, he (ex-boyfriend) may be engaged in fight. The conflict may reach up to lose life. These are some of the problems we expect from improper practicing of ‘Shilshalo’.*

Participant 22

*It was because the way I heard about it was related with ‘Shilshalo’, that was unthinkable to me. As I told you there are some who play ‘Shilshalo’ improperly. So that such persons may be faced pregnancy, later on they said, I had approved my virginity but I gate pregnant. They reason out this as occurred due to ’Shilshalo’. I think this is a pretext they provided to cover what they wrongly did, unless it is difficult to imagine having pregnancy by doing ‘Shilshalo’.*

Participant 23

*When I was at the age of adolescence, I was motivated to play ‘shilshalo’ due to the internal feelings I felt. As that age, I thought every person had feelings that lead them to have something (she bowed her head and laughed). I felt the same, as that time I was in between adolescence feelings, love and keeping my virginity. That means I was in trouble to choose what I should do. In one hand there was love and adolescence feelings, on the other hand there was the issue of virginity. At that time how ‘Shilshalo’ became a solution to overcome such problems, so this was the reason what I motivated to play ‘Shilshalo’.*

Participant 24

*Love coupled with adolescence feeling initiated me to play ‘Shilshalo’. In our culture, being a female mean, you do not have a right upon choosing your future life partner. Our families have decided all about us. As a human being we feel something that brings joy for us. For example, in my case I want to marry the man I loved but my culture does not allow me to do that. So I used ‘Shilshalo’ to get my beloved one before I married someone that I may not like. However, if I make an open sexual intercourse by mistake, I would lose my virginity and this would in turn create different societal problems upon me. Therefore, in order to escape from such bad condition I take care when I perform it.*

Participant 25

*I have played ‘Shilshalo’ when I do not have any option to treat my adolescence feelings. It is a time that I have challenged by being adolescent. Imagine as this time to escape from this problem what I have other than ‘Shilshalo’? When I do not allow marrying, how I can be enjoyed with a girl I loved? However, that can be possible with ‘Shilshalo’ so it give me such opportunity.*

Participant 26

*I did not have understanding about the problem from ‘Shilshalo’ if it was practiced properly, but in the playing place there might be cutting stone or thorn that could be brought some wound on the body, but, this was insignificant. If there was improper playing of ‘Shilshalo’, it might have a great problem. Among these a girl might lose her virginity, so she might be battered by her husband to tell a person who took her virgin. After she told the name of a person, her husband either made a conflict with him (her ex-boyfriend) or he took the issue to ‘abagar’ and forced him to bring compensation.*

Participant 25

“Sometimes I heard it but, I did not believe. How it could be? I think it was a pretext of those who played *‘Shilshalo’* improperly.”

Participant 26

*A fear which I had when I played ‘Shilshalo’ was the case of my virgin. If we had sexual intercourse by mistake, I might lose my virginity and faced very difficult situation. Therefore, in order to escape from such bad condition I took a care when I played it. But I think other problems from thorn, cutting stone, felt down and others couldn’t be taken as a problem. Problems from such things could cure easily as no one knows. These could not make much worry*.

Participant 27

*Injuries like pricked by thorn, amputated by cutting stone, scratched by splinter or bush etc. who took these as a problem? No one considered as harm. These were so simple; they could be cured easily by spilling urine.*

Participant 28

*In the course of playing there could be body injuries or wound on both of us that resulted from felling on unsafe area, but these were not considered as problem.*

Participant 29

*There might be like wound, body injuries etc. from cutting stone, thorn and felt down. However, they couldn’t be taken as problems because problems from such things were cured easily as no one knows, so these wouldn’t make anyone too much worry.*

Participant 30

*When they (boyfriend and his friends) were two and above, how playing of ‘Shilshalo’ was ugly game. Imagine after I had enjoyed pleasantly with my boyfriend, I played with his friends only for the sake of my boyfriend interest. However, the boys were full of energy and eager to play. They did not worry about me. They felt me down hard. They exerted excessive force on me. These all were what I experienced. I faced this situation more than four times in my stay with my boyfriend. As that time I had exhausted. I had felt body fatigue. I hated ‘Shilshalo’ when I played it with my partner’s friends. His friends did not worry about me. They used excessive force upon me and they made me to be weakened. In spite of this hardship, I did not try to refuse this practice because I feared that my boyfriend would be annoyed if I failed to satisfy his friends’ need.*

Participant 31

*That was what I had winced as I remember about my experience of ‘Shilshalo’. As that time I felt tiredness after I had made ‘Shilshalo’ with two or more boys. I had done it in love only with my boyfriend, but with his friends (she thought long---) I played with no feelings. They played only with my body. As a result, they threw down me hard. They did not bother about anything other than their feeling. However, all things they did become bitter for me. I think that make sense for one who experienced it.*

Participant 32

*Sometimes I felt tiredness especially when I played with more than one boy. In that case all what I did except with my boyfriend gave me no sense, so I shared nothing from them except their maltreatment and trouble. I think being tired out in these occasions was one of the ugly parts what I faced in my experience of the practice.*

Participant 33

*Conceiving a child is only needs the coincidence of male sperm cell and female egg cell whether a penis is entered or not inside the vagina. When we see the case of ‘Shilshalo’ a male sperm is sprinkled around the gate of the vagina. That means a sperm cell has possibility to flow into the womb whether she is virgin or not. If that is so conception may be occurred if a girl is at ovulation period, so by doing ‘Shilshalo’ a virgin or a teenage girl may face pregnancy.*

*As long as a male sperm cell is flow around the mouth of the vagina, it has a potential to enter inside of the vagina. As a result it may be fertilized if it gets ready female egg cell whether she is a virgin or not. That means a virgin teen girl may be faced pregnancy due to ‘Shilshalo’ rather with no entering of a male penis inside the vagina.*

Participant 34

*I got a virgin pregnancy when I worked at chira health center in 2003 E.C. A girl was around 15 years old and she came with her mother. Since she feared to tell me what she faced, her mother told me on behalf of her. All the cases she told me about her daughter could be taken as symptom for pregnancy. She also had long time as she experienced menstruation. I ordered her to take a urine exam, however her mother tried to convince me as she was not a pregnant. The mother said me as they approved her virginity by a woman who test virgin of a girl.*

*After all the result showed that she was positive for both i.e. she conceived as the same time she was a virgin. I asked a girl as if she played ‘Shilshalo’ or not. She confirmed me as she had participated in the play. Then I told the result for her mother and this might be due to ‘Shilshalo’. She shocked and entreated me to do all possibility to terminate the fetus from growing. She further explained as the situation made their family dishonored. However, I advised her not to think of any action to do that. Otherwise she lost her daughter life. Later on I heard she made an abortion in Dessie and she was not return back in to the health center.*

*In February 2005 E.C. when I have been working in Senkel health center, a girl came with her mother for contraceptive service since she was at the eve of her marriage. In such case we allowed them to use contraceptive either by sign of menstruation or by checking absence of conception. Fortunately she was not on menstruation so there was a need of pregnancy test. However, the result showed that she was a pregnant. A girl explained she did not make sex but, she had experience of ‘Shlilshalo’. Then virginity test also made to her but the result also indicated she was a virgin. After that I tried to clear them how that could be happened to her due to ‘Shilshalo’ and at the end we reached an agreement as that was not the fault of a girl. I advised them what should be done as a pregnant. Then nothing was known or heard about her.*

*I experienced virgin pregnancy in 2004 E.C. when I worked at Gobera health center. A girl was around 15 years. She came with her mother. As that she was a pregnant of around six months. However, her mother did not think as she became pregnant since she had an approval for her virginity from traditional virginity test practitioner. I gave them explanation regarding the possibility of virgin pregnancy as the like of ‘Shilshalo’ and ordered to take laboratory test.*

*The result showed as she was pregnant and as the same time we also approved her virginity. After this I discussed with her (a girl) about her experience. She told me that as she participated in ‘Shilshalo’ with four or five boys. But, she was unable to identify from whom this problem occurred.*

*Then I dealt with her mother. I warned her not think of termination of the fetus, unless they lost her life. I added the only option they had was try to treat the issue with ‘abagar’, first by doing her father to believe on it. However, regarding her pregnancy no action should be taken against rather she should follow medical treatment and finally she should be delivered at the health center, otherwise her virginity brought complicated problem.*

*After that her father was not willing to accept and brought to ‘abagar’ and she sent to her aunt’s home since her father ordered to leave the home. She had not made any prenatal care.*

*Finally, they brought her at the health center when labor of delivery started.*

*The problem I experienced was pregnancy. First when I felt sick my mother asked me whether I made sexual intercourse or not, I replied that I did not. Then, she took me to a woman who can check the presence or absence of virginity. She approved my virginity. After that we believed it was not pregnancy rather it might be something which could be cured by taking traditional herbs. I took the roots of ‘embuay’ and ‘girawa’, but no improvement was seen. As the time had gone, my belly had grown. After this my mother feared and taken me to Gobera health center. The nurse assured my pregnancy. We told my virginity and asked them how pregnancy could happen in this situation. They also examined my virginity and approved it. After they explained about the possibilities of being pregnant with virginity, I believed it for the first time.*

*After that my mother feared what would be happen on me when my father heard. To make me free from my father’s emotional harm, she preferred to put me with her sister. After that she told him all what occurred. Then he (my father) had brought the issue to ‘abagar’ to negotiate the families of my former boyfriend who impregnated me. Fortunately he denied the case by wanting to take an oath. After that my father ordered my mother as she could not return me back to home. Then my mother dealt with her sister and they decided that I should live with my aunt. Finally I was taken to health center for delivery when the day to give birth reached. At that day I gave birth. After giving birth I was taken to my aunt home. I continued living there with my child. However, my child died after three months and then I returned back to my family.*

Participant 35

*A problem I faced was pregnancy before three years. First when I felt sick I told my mother. My mother said me as it was easy and I would feel better. But, when my abdomen began to grow my mother asked me what I had practiced and who made me pregrant. I replied to her all what I had and I became a virgin. Then she brought me to a woman who tests virginity. She approved the presence of my virgin. After this ‘doqa’ meaning in English preach by elderly men, was made to me. After this my belly pushed forward. Persons who saw me started laughing and pointing their finger at me. After that I started living in the home. I did not want to come out from the home. I forced to stay in the home lonely. When I faced labor of delivery, our village’s traditional birth attendant helped me. But, that was impossible to deliver and finally they brought me to the nearby health center, i.e. Kolegna health center. I delivered a child there. It was died soon as she out from me.*

Participant 36

*A virgin teen age girl having pregnancy mean, she would be a victim of complicated problems. Imagine a girl who participates in this practice is not more than 16 or 17 years. That means she is kid, she is not physically enough matured to give birth. These combined with the community culture worsened the situations of a girl. The community belief inclined to traditional herbs rather than went to health center when they feel sick. Similarly a girl facing pregnancy also tries to treat her sickness in this way rather than checking what happen on her by going in to health center. She remains with no medical treatment.*

*Therefore, the combined effect of her physical immaturity, frequent use of traditional herbs and absence of antenatal treatment bring a problem on the overall health of a teen girl and complicated her labor of delivery. Among these problems some are risk of anemia, hypertension and obstetric risk factors such as urinary tract infection, pelvic cancer and fistula.*

Participant 37

*If a teenage pregnancy occurred, a girl would face problems related with her health and socio-psychological conditions. In the first place, it is difficult to know the occurrence of pregnancy at the early stage. That is due to limited awareness of the community as the possibility of virgin pregnancy and lack of habit to go to health center. So such type of pregnancy could be known after the fetus has grown. That means it reached at the stage of difficulty for termination. Pregnancy by itself especially at age of ‘Shilshalo’, participants arise complicated health problems on the teen mother and new born infants. The mother might be faced a health problems like anemia, hypertension etc. due to absence of prenatal care as well as obstetric related risks such as complexity of delivery labor, urinary tract infection, fistula. However, before all these a girl may be decided to terminate the pregnancy. If they thought termination of the fetus their possibility are undertaking unsafe abortion that can be done by traditional practitioner mostly using of herbs. Thus, performing abortion in this way made a girl to face complicated problems that may reached to loss of life.*

Participant 38

*A girl is more likely to expose for the contracting of STIs than boy. As the male sexual fluids get a chance of entering to the girl’s vagina, transmission of STIs, including HIV/AIDS to the girl is likely to happen. Moreover, one thing is different from the case of pregnancy is to some extent a boy also can be infected. Especially if both of them have a bleeding of wound on their lip and their genital organs, in this case the boy can also be exposed for the transmission of such diseases.*

*If there is a bleeding wound or injuries at the lip or areas around genital organs of the participants, their practicing of ‘Shilshalo’ can expose them to contract STIs including HIV/AIDS. For the girls the possibility of infecting with such disease is so high since the practice entertain sprinkling of a sperm cell at the gate of the vagina that may have a chance to flow inside the vagina. What I said is based on medical principle rather than by actually observing of the case as it is difficult to identify the case unlike the cases of pregnancy*.

Participant 39

*It was better to be salient rather than thinking of what happened on me. It was safe to me if I died. I am pregnant without losing my virginity. But nobody including my parents believes me; they just think I am lying. They took it as a joke and blame me as I did open sexual intercourse while I performed ‘Shilshalo’. They said me “at this time no need of pretext, you should took a care when you played.” They insulted me; they named me as” heavy sleeper”. They said me “as she lived by lifting her legs everywhere, now she tried to take ‘Shilshalo’ as a pretext for her pregnancy*

*If a person saw me, they pointed their fingers, laughed at me and insulted me by veiled. Even if it is not like the past still they point their fingers at me (she thought deeply). I faced so many problems due to this. Now I am not playing as I want. I am not going to a place of song and dance. If I go there, I will face something that make me cringed.*

*My future life will be complicated. I left my education when I faced virgin pregnancy. Regarding married life, no one wants me. Now I am considered as disgraceful, dishonored girl, so who wants this type of person for marriage? The only option I have for my future life would be migration to Djibouti. There are persons who cajole the way to Djibouti. Now I await them to go there.*

*Once my life is damaged, so what did I say? Still I haven’t talked as a human. I haven’t played anything as a young. I have ostracized even by intimate friends. They have pointed their fingers, ridiculed, and laughed at me. The only option I have for my future life is to migrate outside Ethiopia. There are brokers who facilitate outmigration in a hidden way. Now, I am waiting them to go outside Ethiopia.*

Participant 40

*‘Shilshalo’ has been practiced by our community for ages. Our religion forbids any form of sexual practice before marriage. Even any form of relationship between young boys and girls before marriage is considered as a sin. We (religious leaders) have often preached this to our followers but most of the community members are reluctant to behave as our teaching.*
